# News and Events of the Week

**Published:** 1911-01-07

**Authors:** 


### News and Events of the Week.

Mr. Robert William Coe, of Pembroke Road, Clifton, Bristol, surgeon, the oldest Fellow of the Royal College of Surgeons of England, who died on August 11, aged eighty-nine, left estate valued at £28,612 gross, with net personalty £27,485.

H.I.M. the German Emperor has been made honorary M.D. of Prague. The last honorary M.D. of Prague was the late Professor Virchow. The Kaiser's interest in medical science, which he has shown in many practical ways, is well known.

The Society for the Study of Inebriety.—The next meeting of the Society will be held in the rooms of the Medical Society of London, 11 Chandos Street, Cavendish Square, W., on Tuesday, January 10, 1911, at 4 p.m. The president of the Society, Dr. Hyslop, will open a discussion on "The Influence of Parental Alcoholism on the Physique and Ability of Offspring." Each member and associate is at liberty to introduce a visitor.

The Presidents of the Royal Medical Society of Edinburgh have issued invitations to a dinner on February 15 to meet the Right Hon. Sir Robert Bannatyne Finlay, M.D., K.C., M.P., and Sir James Crichton-Browne, M.D., LL.D., F.R.S., on the 50th anniversary of their joining the Society while students at the University of Edinburgh.

We understand from the Dowager Lady Longford and Mrs. Alfred Ker that some of the friends and grateful patients of the late W. H. C. Staveley, F.R.C.S., are anxious to communicate with others who may wish to join in doing honour to his memory. To any who care to write, asking for information, further details will be gladly sent by Captain the Hon. Edward Pakenham, 24 Bruton Street, W.; or Miss A. Gladstone, 28 Eccleston Street, S.W.

Princess Louise (Duchess of Argyll), who is patroness of the Kensington Dispensary, on Tuesday last presented a cheque (to be followed later by an illuminated address) to Mr. Ernest H. Bonney, M.R.C.S., L.R.C.P., on his retirement from the dispensary after nearly nine years as resident medical officer. The presentation was made at a concert at the Kensington Town Hall, attended by the Mayor of Kensington (Sir Walter Phillimore) and other leading residents of the borough.

Some of the City Companies, recognising the great value of the public work done by the Royal Society of Medicine, have made grants in aid of the building fund which is being raised by the Fellows to supply the Society with a new house more adequate to its increasing work. Owing to the generosity of various subscribers, prominent among whom are several of the City Companies, there is reason to hope that the building, which is now being built at the corner of Wimpole Street and Henrietta Street by Mr. John Belcher, R.A., may be opened free from debt on account of this exceptional expenditure for a new site and house. We understand that the normal income of the Society, provided by the Fellows, is quite sufficient for carrying on the Society's public and special work, and the Council is anxious that this shall not have to be curtailed because of any charges on account of the new building.

Lord Warwick, as Lord Lieutenant of the county, presided at a meeting of the Essex County Association formed to combat consumption, held at Chelmsford. He proposed that a memorial be raised to King Edward, and that it take the form of something to combat the scourge of consumption. This was adopted. He added that he had accepted an offer from Mr. Boyd, of Sandon, of a house and site at Little Gibcracks, Baddow, and also £800 towards its maintenance.

The Cartwright Prize offered by the Association of the Alumni of the College of Physicians and Surgeons, Columbia University, New York, is open for universal competition, and will be awarded in 1911. The amount of the prize is $500. The essays submitted in competition must contain original investigations made by the writer, and must be the work of only one person; they must be typewritten, in English, marked with a device or motto, and accompanied by a sealed envelope, similarly marked, containing the name and address of the author. Essays should be sent to Dr. H. E. Hale, Secretary, 770 West End Avenue, New York, on or before April 1, 1911.

A Sessional Meeting of the Royal Sanitary Institute will be held in Bradford on Friday, February 3; the members will meet at the Town Hall, Bradford, at 2.30 p.m., and proceed to visit the Open Air School, Thackley, by special tramcar; at 7.30 p.m. discussion will take place in the Council Chamber, Town Hall, on "School Clinics," to be opened by L. A. Williams, M.D., D.P.H., Medical Superintendent Bradford Education Authority. The following have consented to take part in the discussion: J. R. Kaye, M.B., C.M., D.P.H., M.O.H. West Riding; S. G. Moore, M.D., Ch.B., D.P.H., M.O.H. Huddersfield; E. M. Smith, M.D., C.M., D.P.H., Medical Officer of Health, York; A. E. L. Wear, M.D., M.B., B.S., School Medical Officer, Leeds; Ralph P. Williams, M.D., B.S., D.P.H., School Medical Officer, Sheffield. General discussion is invited. The chair will be taken at 7.30 p.m. by Louis C. Parkes, M.D., D.P.H. Tickets for admission of visitors may be had on application to Dr. W. Arnold Evans, Medical Officer of Health, Health Department, Town Hall, Bradford, who is kindly acting as the local hon. secretary of the meeting.

"The Prescriber."—The fourth volume of this interesting and valuable little journal—which is one of the most useful publications the practitioner can subscribe to—contains a mass of information. The exhaustive index, which is a feature of the yearly volume, makes it easy to look up any particular subject or prescription. And there are many subjects discussed in this volume (which is, of course, the various monthly numbers bound up—and very neatly bound up, too—in the yearly volume cover). Among these subjects we have, for example, the one which has yielded so much food for debate recently—the merits of 606. The prescribing notes will be specially appreciated by practitioners who have to dispense their own medicines. The main feature of "The Prescriber" is that its information is throughout practical and useful. There is not a page of useless knowledge in the whole volume; there are many pages of sterling worth, which give the book a permanent value. Medical men who do not yet know the little monthly should make its acquaintance. They can easily do so by writing for a specimen number to the offices, 137 George Street, Edinburgh.

Last week an audience of 300 or 400 saw the performance of *The Kiss*, by "George Paston," in which the Duchess of Westminster represented the principal character, Mistress Chalmers. She was assisted by Mrs. Fitzpatrick as Mrs. Budgen, Mr. J. Cornwallis-West as the Stranger, and Mr. N. Forbes-Robertson as Mr. Wharton. The play formed a part of a miscellaneous entertainment organised by the Duchess in aid of the funds of the Royal Alexandra Hospital, Rhyl. The performance was given in the library, and the company included Katharine Duchess of Westminster, Lord and Lady Crichton, and Lady Helen Grosvenor.

The following are the arrangements for next week at the Medical Graduates' College and Polyclinic, 22 Chenies Street, Gower Street, W.C. : Tuesday, January 10, at 4 p.m., Dr. Harry Campbell, Clinique (Med.); at 5.15 p.m., Dr. C. O. Hawthorne, "Instrumental Methods in the Estimation of the Arterial Blood Pressures." Wednesday, at 4 p.m., Mr. R. P. Rowlands, Clinique (Surg.); at 5.15 p.m., Dr. R. W. Allen, "Vaccine Treatment in Pulmonary Phthisis." Thursday, at 4 p.m., Dr. Eric Pritchard, Clinique (Med.); at 5.15 p.m., Dr. S. E. Dore, "The Present Position of the *x*-Ray Treatment of Ringworm of the Scalp." Friday, at 4 p.m., Mr. Leslie Paton, Clinique (Eye).

The following are the arrangements for next week at the West London Postgraduate College : At 10 a.m., January 9 and 12, demonstration by Surgical Registrar; at 10 a.m., January 13, demonstration by Medical Registrar; at 11.30 a.m., January 10, demonstration of minor operations; at 12 noon, January 9, pathological demonstration; at 12 noon, January 12, lecture by Dr. Grainger Stewart on Practical Medicine. Lectures at 5 p.m. : January 9, Mr. Etherington-Smith, Clinical lecture; January 10, Dr. Low, "Plague"; January 11, Dr. Morton, "Carbon Dioxide"; January 12, Mr. Baldwin on Practical Surgery; January 13, Dr. Saunders, Clinical lecture with cases.

